# Scalable Analysis of Untargeted LC-HRMS Data by Means of SQL Database Archiving

**DOI:** 10.1021/acs.analchem.2c03769

**Published:** 2023-02-20

**Authors:** Marie Mardal, Petur W. Dalsgaard, Brian S. Rasmussen, Kristian Linnet, Christian B. Mollerup

**Affiliations:** † Department of Forensic Medicine, University of Copenhagen, Frederik V’s vej 11, Ø Copenhagen, Denmark; ‡ Department of Pharmacy, The Arctic University of Norway, Hansine Hansens veg 18, 9019 Tromsø, Norway

## Abstract

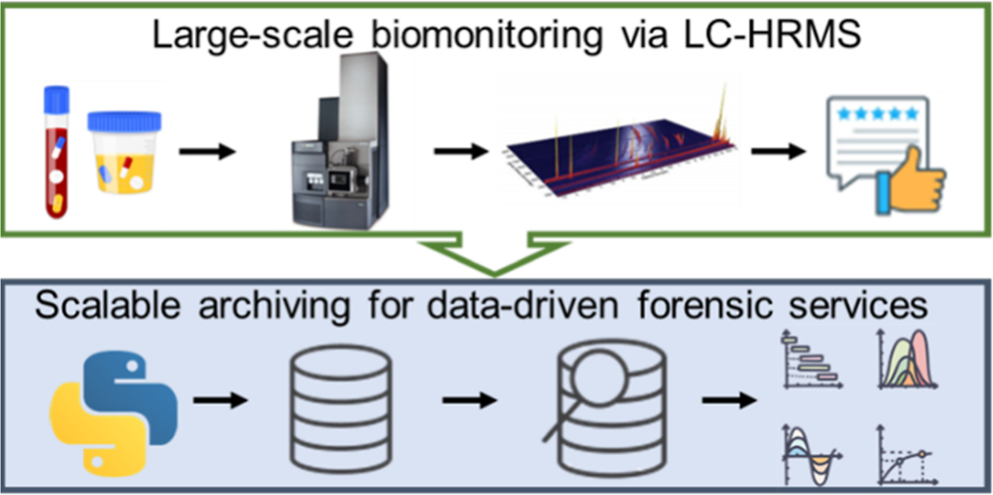

Liquid chromatography-high-resolution mass spectrometry (LC-HRMS) is widely used to detect chemicals with a broad range of physiochemical properties in complex biological samples. However, the current data analysis strategies are not sufficiently scalable because of data complexity and amplitude. In this article, we report a novel data analysis strategy for HRMS data founded on structured query language database archiving. A database called ScreenDB was populated with parsed untargeted LC-HRMS data after peak deconvolution from forensic drug screening data. The data were acquired using the same analytical method over 8 years. ScreenDB currently holds data from around 40,000 data files, including forensic cases and quality control samples that can be readily sliced and diced across data layers. Long-term monitoring of system performance, retrospective data analysis for new targets, and identification of alternative analytical targets for poorly ionized analytes are examples of ScreenDB applications. These examples demonstrate that ScreenDB makes a significant improvement to forensic services and that the concept has potential for broad applications for all large-scale biomonitoring projects that rely on untargeted LC-HRMS data.

## Introduction

Liquid-chromatography-high-resolution mass spectrometry (LC-HRMS) is widely used for comprehensive screening of complex samples in environmental,^1^ forensic,^2^ clinical,^3^ and food^4^ chemistry. The screening is considered comprehensive because it can cover chemicals with a wide range of physiochemical properties and allows the use of a flexible target list since data acquisition typically occurs in an untargeted manner. The acquired data set is rich, with each analytical compound identified as an *m*/*z*-retention time pair, possibly supported by additional parameters such as the fragment ions, adduct pattern, or isotopic pattern. LC-HRMS data originally acquired for one purpose is reanalyzed to answer new research questions using retrospective screening,^5−7^ metabolomics applications,^8−11^ and nontargeted screening.^2^ The data files are mostly queried at the batch level using vendor or open-source software via open file formats. The data analysis strategies for re-use of >1000 LC-HRMS data files typically involve either significant data reduction or are limited to the original research question, thereby requiring process-heavy reanalysis for new questions. This article reports a novel data analysis strategy for LC-HRMS data that involves the additional storage of peak deconvoluted data in a structured query language (SQL) database format, allowing for quick reanalysis to answer new research questions.

Untargeted and unannotated analytical data from our LC-HRMS forensic drug screening collected in 8 years was parsed to an SQL database, referred to as ScreenDB. Forensic analyses adhere to strict quality assurance schemes to ensure the traceability and reproducibility of results. After a method development, validation, and implementation phase, a screening workflow will run with only minor modifications resulting in data that is comparable over time. ScreenDB was set up with the objective of linking the screening data with other forensic metadata, and the database should allow the access to all relevant data layers including adduct, isotope, and fragment ions. Our applications of ScreenDB show functional data workflows with >10,000 LC-HRMS data files.^8,12,13^ This novel data analysis strategy for LC-HRMS data is scalable to at least 40,000 data files, although only the server hardware theoretically sets the limit. SQL archiving is thus ideal for active storage of large amounts of comparable LC-HRMS data sets if the data owner wishes to frequently query data.

## Experimental Section

### Instrumentation

The sample preparation, LC-HRMS settings, and subsequent data evaluation workflow were previously described by Mollerup et al.^2^ Briefly, chromatographic retention was performed with reversed-phase LC in the gradient mode with a total run time of 15 min. Data were acquired on three different Xevo G2-S QTOF HRMS instruments (Waters, Milford, USA) with comparable parameters and sensitivities, in the MS^E^ data-independent acquisition mode.

### Drug Screening

Internal standards were added to all samples prior to sample preparation. Within-run quality control (QC) samples included blank matrices, internal-standard blank injections, and injection of three methanolic standard mixtures, each containing approximately 100 compounds at 0.5 mg L^–1^. The quality of every batch and injection was verified as part of routine forensic data analysis. Only data from analytical runs that fulfilled set forensic QC criteria were transferred to the database. All data was analyzed in UNIFI instrument software (Waters, Milford, USA) and then exported as the UNIFI export package format (.uep). Since the data was previously used in forensic drug screening workflows, data files were reprocessed to remove annotations and to lower the ion count threshold. The count threshold was lowered to allow evaluation of these set thresholds.

### Data Analysis

LC-HRMS measurement variables were read directly from the uep data files and subsequently parsed to the SQL archive. To prepare plots, data were extracted from ScreenDB with the SQL server (Microsoft, Redmond, Washington, USA), and subsequent analysis steps were made using Python. Data variables of internal standards for all biological samples and selected QC analytes analyzed between 2014 and 2020 were extracted from ScreenDB with one diagnostic fragment ion with precursor ion limits of exact mass ± 3 mDa. These ions were then grouped using the mean-shift clustering algorithm from the SciKit-learn Python package. All peaks were scaled using tolerances of 3 mDa and 0.5 min between samples. The mean-shift clustering algorithm was subsequently applied using a bandwidth of 1. Diagnostic fragment ions were grouped if present at 0.015 min from the precursor ion.

## Results and Discussion

### ScreenDB Architecture and Content

**Figure 1:**
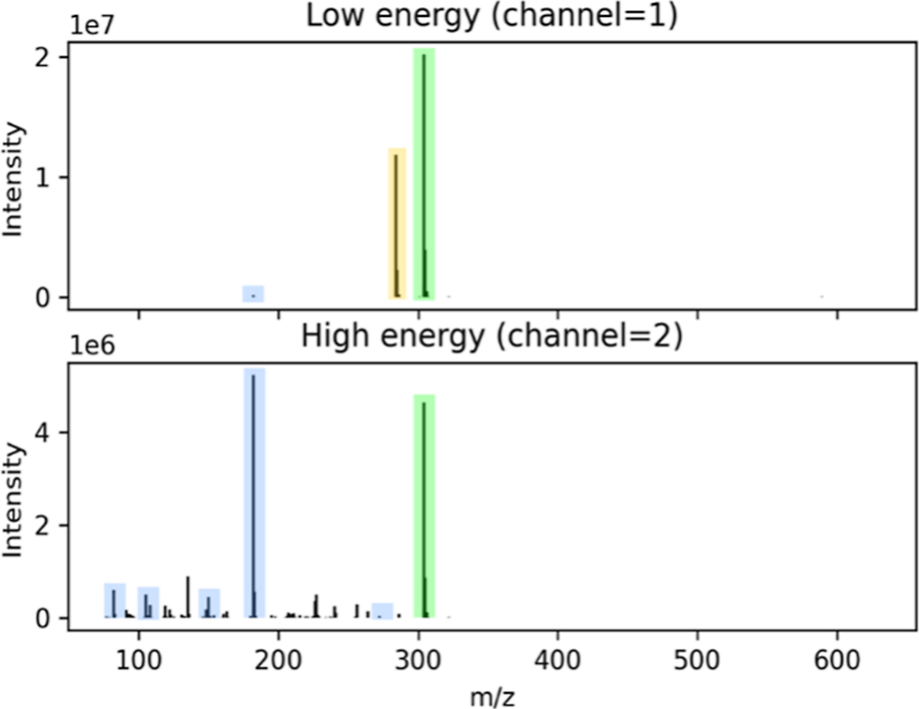
Low- and high-energy spectra extracted from ScreenDB for cocaine in a methanolic QC. Green boxes: [M + H]^+^ and residual [M + H]^+^ with isotopic patterns in low- and high-energy spectra, respectively, blue boxes: *in-source* fragment ion and diagnostic fragment ions in low- and high-energy spectra, respectively, and yellow box: contaminant ion.

Different types of forensic cases were screened with the same LC-HRMS method, including driving-under-the-influence-of-drugs, drug seizure, biological samples from autopsy, and drug-facilitated crime cases. Registration and documentation are handled through a laboratory information management system, STARLIMS (STARLIMS Corporation, Hollywood, FL, USA). The sample name, analytical run number, and analysis identifier nomenclature in ScreenDB refer to the entries in the LIMS. Via this structured connectivity, data from each LC-HRMS injection can be matched with well-curated data consisting of quantitative results from complementary methods, the sample age, case characteristics, and other historical data. ScreenDB consists of two tables, with Tables S1 and S2 presenting the most important variables parsed from the uep files. A sample table holds sample-specific information, such as the sample identifier, file directory, run name, and a unique data identifier (uid) assigned to the raw data using vendor software. Each analytical injection corresponds to one line in the sample table. In the peak table, each line corresponds to one ion signal from either low- or high-energy channels. Each signal has an accurate mass, retention time, and signal intensity, together with diagnostic variance parameters calculated in the compression (componentization) process from the profile data. The UNIFI componentization step involves peak detection and subsequent grouping of co-eluting ions in the high- and low-energy spectra,^14^ and thus filtering out of some background signals that do not show chromatographic retention. The precursor ion, *in-source* fragment ions, isotopes, and adducts are available from the low-energy spectra, and the residual precursor ion and fragment ions are available from the high-energy spectra, along with some co-eluting interferences. Ions are decomponentized in ScreenDB, meaning that each measured ion at a given retention time results in a line in the peak table, and it can be queried independently from the assigned component. Variables available from the ScreenDB peak table are illustrated in Figure 1 for the drug cocaine. The spectra show how isotopic peaks, diagnostic fragment ions, and protonated molecules are available for queries. Extracted spectra for cocaine and morphine are available in Figures S1A and S2A, respectively, from the lock-mass corrected and centroided raw files and from the uep files from the same injection as that in Figures 1 and S2B to illustrate data retention in the componentization step. Complex, multiparametric queries are not easy to build in vendor software but can be coded in general-purpose programming languages with extracts from ScreenDB. A legacy version of ScreenDB used for earlier applications^8,13^ was based on mascot generic format files that associated each low-energy ion with the component’s high-energy spectrum. This inflated the legacy ScreenDB and also required conversion of the data files prior to storage. Reading the uep files directly allows the retention of more analytical information and access to the extra data layers arising from profile acquisition. The use of the mascot generic format in combination with MS^1^-level features is used in feature-based molecular networking, where only representative MS^2^ spectra are selected to eliminate this data inflation.^15^ Feature-based LC-HRMS data with linked fragment ion spectra could work well in an SQL database structure, although ions recorded in low and high energy would have variable levels of information. The database architecture would have to be modified accordingly and may need separate tables for features and fragment ions. Other acquisition and processing software or peak-picking algorithms provide different data variables available for parsing. Without the UNIFI componentization step, another lock-mass correction and peak-picking algorithm should be used to centroid ions in the mass and retention time domains. It is, however, crucial for retrospective and non-targeted data analysis workflows that the data layers remain accessible separately for flexible queries that are not locked in feature formats. Our purpose of making this structured digital archive was to mirror and support the in-house screening, which uses UNIFI peak components, and therefore, alternative approaches were not evaluated as this would not fulfill our research goals.

### Measurement Uncertainty

**Figure 2:**
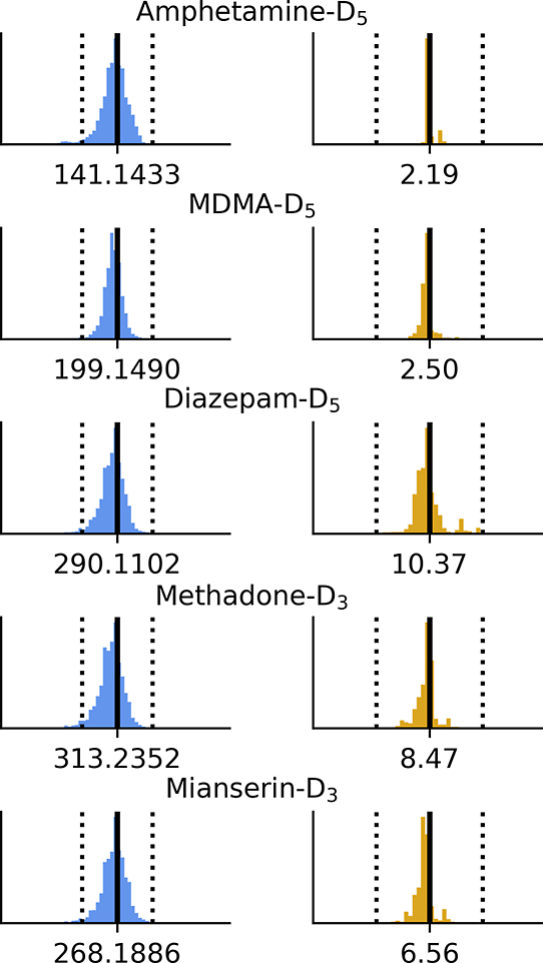
Measurement uncertainties in the mass (blue) and retention time (yellow) domains for internal standards measured in biological samples over 7 years of analysis. Amphetamine-*d*_5_ was replaced with MDMA-*d*_5_ in 2017. Solid lines indicate library values, and dotted lines indicate 1 mDa and 0.25 min windows.

**Figure 3:**
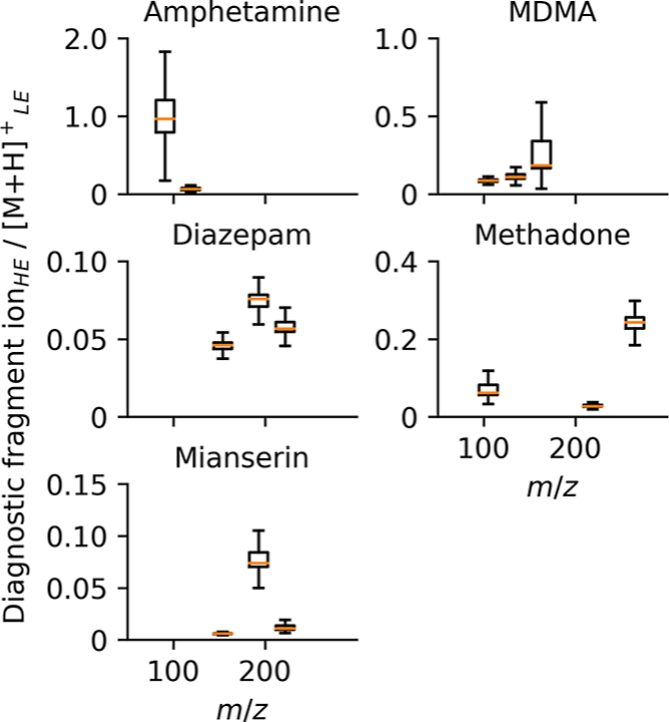
Measurement uncertainty of fragmentation for five reference standards in methanolic QC samples over 7 years of acquisition. HE: high-energy channel and LE: low-energy channel.

Reproducible measurements are imperative for the meaningful comparison of data variables acquired over years of analysis. A strength of ScreenDB is that stored QC sample variables and internal standard signals are accessible for further quality check and to set informed limits. Histograms of the protonated molecules in the mass and retention time domains from internal standards in around 14,000 biological samples are presented in Figure 2. Fragmentation reproducibility is presented in Figure 3, with 5 standards from around 1000 methanolic QC sample injections. When performing big data analyses with ScreenDB, the extracted ion chromatograms and fragment ion spectra are not evaluated individually, as opposed to forensic screening evaluation. Therefore, well-informed and more tailored decisions need to be made, as presented in Pan et al.^12^

### Applications of ScreenDB

#### System Monitoring

**Figure 4:**
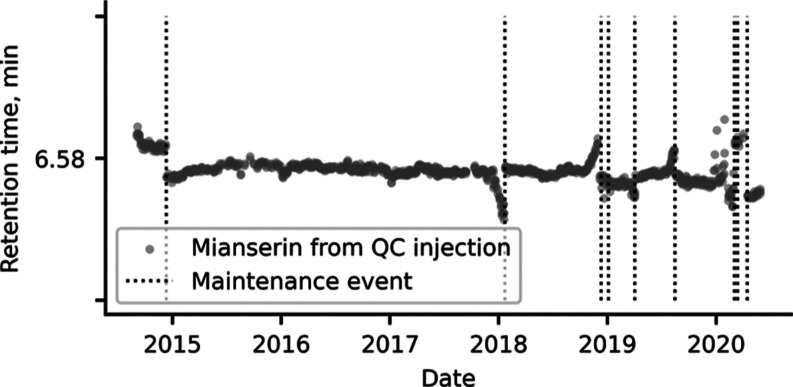
Mianserin retention time from a methanolic QC injection from ScreenDB extracted together with logged maintenance events initiated in the LIMS from leaks or analytical column change. The *Y*-axis represents target library retention time ±0.5 min.

In accredited analytical laboratories, systems are regularly maintained and evaluated with system suitability testing. Monitoring of system performance with ScreenDB variables supports informed decisions during troubleshooting sessions, as exemplified in Figure 4 with data from a single LC-HRMS instrument. These plots can help chemists distinguish between insignificant drifts and problems needing intervention, when combined with logged instrument maintenance events.

#### ScreenOmics

**Figure 5:**
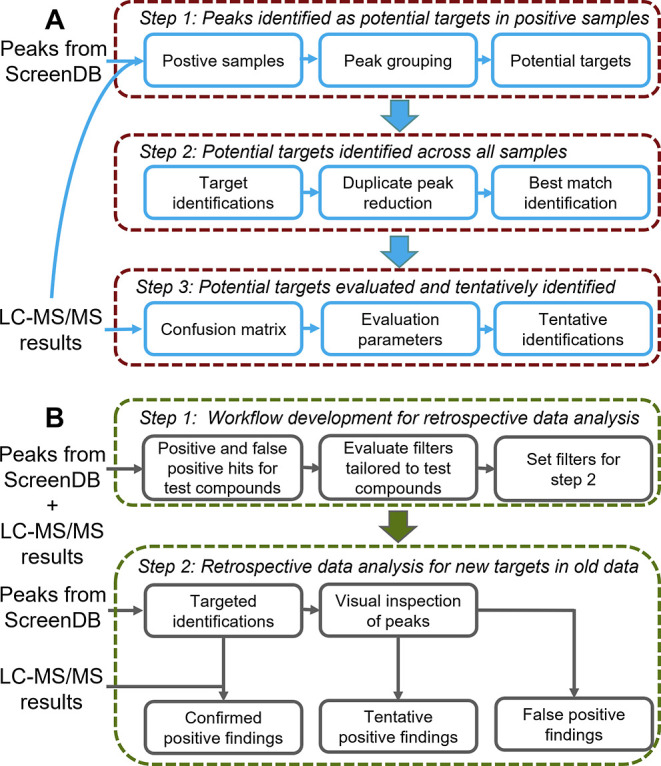
ScreenDB data analysis workflows: (A) ScreenOmics for identification of alternative screening targets. (B) Workflow for retrospective data analysis for new targets in old data files.

The in-house drug screening workflow is improved by identification of new targets for analytes that cannot otherwise be detected via metabolomics-type workflows.^8,13^ A toxicological screening is frequently carried out with positive electrospray ionization and therefore has lower sensitivity for neutral and acidic compounds. Comparing the LC-HRMS data from samples with known positive and negative quantitative results for a given drug allows the identification of alternative targets (Figure 5A). This is referred to as ScreenOmics. The ScreenOmics approach does not use signal intensities for prioritization of targets but instead takes advantage of the large number of control samples accessible in ScreenDB. Alternative targets may be adducts and/or metabolites that ionize better in positive electrospray ionization than the parent compound, as described for valproate^8^ and barbiturates.^13^ These workflows are only possible because all ions associated with a feature are accessible in ScreenDB.

#### Retrospective Data Analysis

When a new drug emerges, ScreenDB can be queried to disclose whether a feature with those characteristics has ever been acquired. Retrospective data analysis is performed in a matter of seconds, making this approach for retrospective screening more efficient than reprocessing data or reanalyzing samples. Recently, 13,514 data files from driving-under-the-influence-of-drugs samples stored in ScreenDB were queried for designer drugs.^12^ A data workflow was tailored to common benzodiazepines using quantitative results from a complementary analytical method as a true condition (Figure 5B). This study revealed 43 tentative positive findings that were not detected in the screening when the case was open and only 9 false positive findings.

Feature-based data analysis can answer many of the same and more research questions than ScreenDB but can only scale to 2000 samples by limiting the number of features for downstream analysis.^15^ Vendor software used for the drug screening workflow in our laboratory becomes slow in batches of >100 samples, and ion signal data is locked in components. ScreenDB was therefore developed as a structured library of ion signals to enable active reuse of LC-HRMS data and flexible access to available data layers. The scalability and flexibility of LC-HRMS^E^ data analysis via SQL database archiving are not achievable with other platforms. Moving data to an SQL archive is an alternative to relying on memory upgrades to run larger batches. When the ion signals are archived as tabular data, the data can no longer be directly imported in the vast number of computational tools available for LC-HRMS data analysis workflows. Consequently, data analysis workflows need to be developed de novo, and some programming is necessary to make use of the data. Storing data in the SQL format increases storage space demands and most often requires a separate database server. However, the price of the necessary hardware or cloud services is minuscule compared to the price of the LC-HRMS hardware.

## Conclusions and Perspectives

In this study, we report a novel scalable strategy for LC-HRMS data analysis. ScreenDB is an SQL database that currently stores data from around 40,000 data files, acquired with a single analytical method from 2014 onward. ScreenDB can be used as a stand-alone data source, but its main value for forensic toxicology lies in the linking with digitalized laboratory and case data. In our laboratory, we frequently query the database for contaminant trouble shooting and retrospective data analysis and to improve our drug screening method. Easy access to historic data is a prerequisite for it to be of any value in high-throughput laboratories. Being an SQL database, we only have to connect with ScreenDB (<1 min), and then, we can query 8 years’ worth of LC-HRMS data with the same level of information available in vendor software but with both flexibility and speed. Scalable data analysis approaches as presented here are necessary as large-scale biomonitoring with LC-HRMS becomes more prevalent. Active storage in SQL databases can expand the impact of large-scale biomonitoring projects by making the data more accessible, reusable, and interoperable. However, robust analytical systems that are evaluated with QC systems are imperative to compare data over long periods of time.

Readily retrievable, curated, and untargeted analytical data enable fast and simple retrospective analyses for new targets and active use of stored intelligence in historic LC-HRMS data. Consequently, transferring data to a structured digital archive augments active data reuse and, in our case, improves forensic services.

## Supporting Information Available

Example sample data table from ScreenDB, example peak data table from ScreenDB, and extracted spectra of cocaine and morphine in a QC injection before and after componentization (PDF)

## Notes

The uep file reader code is publicly available in a GitHub repository at https://github.com/ucph-rka/ScreenDB together with a Jupyter notebook which demonstrates how to parse uep data to a sqlite database and query the database to visualize spectral data. ScreenDB is based on samples originating from forensic casework. Therefore, the database is not made publicly available, and confidentiality of sensitive health-related data is maintained.
