# Supplementary material for: Scalable Analysis of Untargeted LC-HRMS Data by Means of SQL Database Archiving
Source: Anal Chem. 2023 Feb 20;95(10):4592–6. doi: 10.1021/acs.analchem.2c03769 (PMC10018448; doi:10.1021/acs.analchem.2c03769)

Supporting information for

Scalable analysis of untargeted LC-HRMS data by means of SQL database archiving

Marie Mardal[1,2], Petur W. Dalsgaard[1], Brian S. Rasmussen[1*], Kristian Linnet[1], Christian B. Mollerup[1]

AUTHOR ADDRESS: [1]Department of Forensic Medicine, University of Copenhagen, Frederik V's vej 11, 2100 Copenhagen O, Denmark. [2]Department of Pharmacy, the Arctic University of Norway, Hansine Hansens veg 18, 9019 Tromsø, Norway.

Table of Content

Table SI1: Sample table from ScreenDB.. **Error! Bookmark not defined.**

Table SI2: Peak table from ScreenDB... **Error! Bookmark not defined.**

Fig. SI1A: Low and high energy spectra of cocaine from lock-mass corrected and centroided raw file 3

Fig. SI2A: Low and high energy spectra of morphine from lock-mass corrected and centroided raw file... 4

Fig. SI2B: Low and high energy spectra of morphine read from uep file 4s

Table SI1: Sample table of selected variables from the ScreenDB with data examples. Columns were named in laboratory jargon and have been given more rational names in the uep reader.

import_id	sample_id	sample	run_name	system_name	uep_path	sample_uid
1	1	Q00189 03100X	20170427 RN-XXXX Screen Fæld Analyse Pos Loke PWD	Loke	\\prod-5685\E\CBM\UnifiData\AutoExport\20170427 RN-39275 Screen Fæld Analyse Pos Loke PWD.uep	9648e2f8b5cd4eb58 3ed47423f49dd7c
1	2	Q00189 03101X	20170427 RN- XXXX Screen Fæld Analyse Pos Loke PWD	Loke	\\prod-5685\E\CBM\UnifiData\AutoExport\20170427 RN-39275 Screen Fæld Analyse Pos Loke PWD.uep	598b7952c895482b9 b2f2f1c3dab2c41
1	3	Q00189 03102X	20170427 RN- XXXX Screen Fæld Analyse Pos Loke PWD	Loke	\\prod-5685\E\CBM\UnifiData\AutoExport\20170427 RN-39275 Screen Fæld Analyse Pos Loke PWD.uep	d86ae7b6fba74579b 177f8fda7f31e6a
1	4	Q00189 03103X	20170427 RN- XXXX Screen Fæld Analyse Pos Loke PWD	Loke	\\prod-5685\E\CBM\UnifiData\AutoExport\20170427 RN-39275 Screen Fæld Analyse Pos Loke PWD.uep	983a2b354522483fa 6c3cd5e3fe3bce5
1	5	Q00189 03104X	20170427 RN- XXXX Screen Fæld Analyse Pos Loke PWD	Loke	\\prod-5685\E\CBM\UnifiData\AutoExport\20170427 RN-39275 Screen Fæld Analyse Pos Loke PWD.uep	c3d19c56b26b420da d5b59eeaf756fb9

Table SI2: Peak table of selected variables from the ScreenDB with data examples. Columns were named in laboratory jargon and have been given more rational names in the uep reader.

sample_id	channel	chargeClusterID	massPeakID	mass	massSD	rt	retentionTimeSD	intensity	intensitySD	counts	countsSD	retentionTimeFWHM	liftOffRT	infUpRT	infDownRT	touchDownRT
1	1	304	1	279.0934	0.000186	0.204	0.000166	88542.4	723.7	10604.0	103.0	0.03839144	0.176	0.187	0.216	0.227
1	1	149	3	279.0934	0.00018	0.402	0.000307	208411.5	1110.2	24959.8	158.0	0.1154382	0.287	0.347	0.430	0.441
1	1	492	4	279.0932	0.000216	0.514	0.000142	26001.9	392.2	3114.0	55.8	0.02009332	0.494	0.504	0.530	0.541
1	1	489	5	279.093	0.000216	0.587	0.000284	27154.0	400.7	3252.0	57.0	0.03812782	0.548	0.559	0.597	0.609
1	1	419	6	279.0934	0.000196	0.640	0.000228	44345.2	512.1	5310.9	72.9	0.03914473	0.608	0.619	0.665	0.681

Fig. SI1: Plots illustrating data retained after the UNIFI componentization for cocaine in a methanolic system control injection. A) Cocaine in a centroided and lock-mass corrected waters raw files, without blank correction. Rather than extracting ions associated with the cocaine component, the ions eluting in a defined time range are extracted for better comparison. Low and high energy spectra from the same sample read from the uep file is available as Fig. 1 in the main text. The system control sample (methanolic) uep file is available together with the uep_reader from the GitHub repository https://github.com/ucph-rka/ScreenDB

A) Cocaine in low and high energy mass spectra (4.50-4.56 min) from centroided, lock-mass corrected waters raw files. Green boxes: $[M+H]^+$ and residual $[M+H]^+$ in low and high energy spectra, respectively, including isotopic pattern, Blue boxes: in-source fragment ion and diagnostic fragment ions in low and high energy spectra, respectively, Yellow box: Contaminant ions

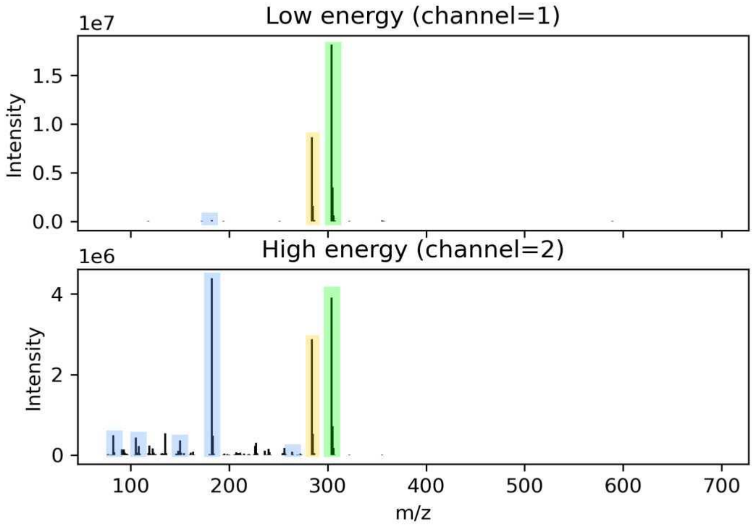

Fig. SI2: Plots illustrating data retained after the UNIFI componentization for morphine (early elution) in a methanolic system control injection. A) Morphine in a centroided and lock-mass corrected waters raw files, without blank correction. B) Morphine extracted in the same data file after UNIFI componentization. Rather than extracting ions associated with the morphine component, the ions eluting in a defined time range are extracted for better comparison. The system control sample (methanolic) uep file is available together with the uep_reader from the GitHub repository: https://github.com/ucph-rka/ScreenDB

A) Morphine in low and high energy mass spectra (1.05-1.08 min) from centroided, lock-mass corrected waters raw files. Green boxes: $[M+H]^+$ and residual $[M+H]^+$ in low and high energy spectra, respectively, including isotopic pattern, Blue boxes: in-source fragment ion and diagnostic fragment ions in low and high energy spectra, respectively, Yellow box: Contaminant ion, Purple box: $[M+Na]^+$ and $[M+K]^+$

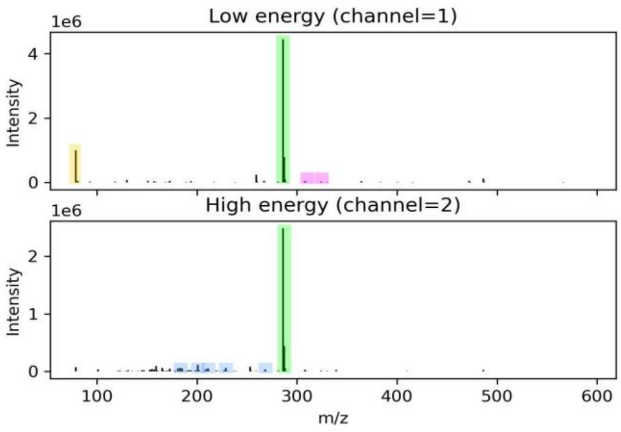

B) Morphine in low and high energy mass spectra (1.05-1.08 min) from uep data. Green boxes: $[M+H]^+$ and residual $[M+H]^+$ in low and high energy spectra, respectively, including isotopic pattern, Blue boxes: in-source fragment ion and diagnostic fragment ions in low and high energy spectra, respectively, Purple box: $[M+Na]^+$ and $[M+K]^+$

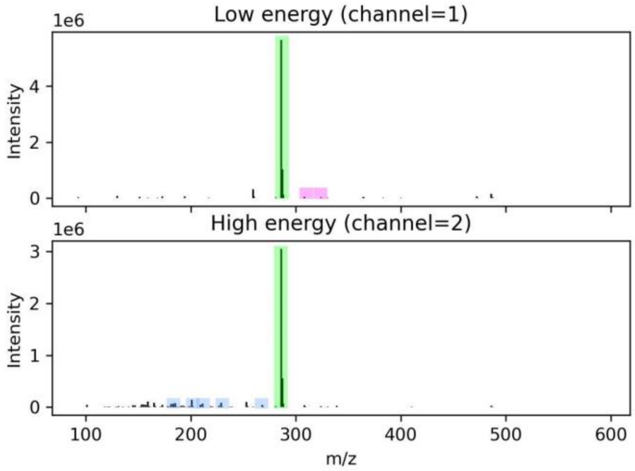

Supplement: Supplementary file 1 — ac2c03769_si_001.pdf [file ac2c03769_si_001.pdf]
